# Diabetes mellitus and mortality in patients admitted to ICU with sepsis: a meta-analysis

**DOI:** 10.3389/fmed.2026.1743706

**Published:** 2026-01-21

**Authors:** Hong Zheng, Feiyong Yu, Chaoyong Bei, Yitong Zhou, Zhongcheng Mo, Bing Wei

**Affiliations:** 1 Department of Emergency Medicine, The First Affiliated Hospital of Guilin Medical University, Guilin, Guangxi, China; 2 Guangxi Key Laboratory of Diabetic Systems Medicine, Guilin Medical University, Guilin, Guangxi, China; 3 Department of Trauma and Extremity Orthopedics, The First Affiliated Hospital of Guilin Medical University, Guilin, Guangxi, China; 4 Department of Dermatology, The First Affiliated Hospital of Guilin Medical University, Guilin, Guangxi, China; 5 Department of Geriatrics, The First Affiliated Hospital of Guilin Medical University, Guilin, Guangxi, China

**Keywords:** diabetes, ICU, Meta-analysis, mortality, Sepsis

## Abstract

**Background:**

Diabetes mellitus (DM) is relatively common among patients with sepsis, yet its precise impact on mortality risk remains unclear. This study aims to explore the relationship between DM and mortality risk in sepsis patients by synthesizing existing data, thereby providing evidence for clinical management.

**Methods:**

A systematic search of PubMed, Embase, Web of Science, and Cochrane Library databases from their inception to October 20, 2025, identified observational studies evaluating the association between DM and mortality in patients with sepsis. A random-effects model was used to pool relative risks (RR) and 95% confidence intervals (CI) to assess the relationship between DM and sepsis-related mortality risk. Sources of heterogeneity were explored through sensitivity and subgroup analyses, and publication bias was assessed using the Egger test.

**Results:**

A total of 13 studies (*n =* 1,209,263) were included in the analysis. The results of the meta-analysis indicate that DM is associated with an increased risk of mortality in sepsis patients [RR = 1.25, 95% CI (1.14, 1.38)]. Subgroup analyses revealed that DM is linked to a higher risk of in-hospital mortality [RR = 1.60, 95% CI (1.11, 2.31)], with stronger associations observed in specific regions and study designs. Notably, studies conducted in China [RR = 1.79, 95% CI (1.19, 2.70)], the Netherlands [RR = 1.14, 95% CI (1.12, 1.17)], and Israel [RR = 1.81, 95% CI (1.55, 2.12)], as well as cohort studies [RR = 1.25, 95% CI (1.13, 1.38)], showed more pronounced effects. Additionally, analyses based on the Sepsis-3 criteria also demonstrated a significant association between DM and increased mortality risk [RR = 1.59, 95% CI (1.24, 2.04)].

**Conclusion:**

This meta-analysis indicates that DM is significantly associated with an increased risk of mortality in patients with sepsis, particularly with respect to in-hospital mortality. The observed association may be partially explained by DM-related pathophysiological mechanisms, such as immune dysfunction, dysregulated inflammatory responses, and the presence of chronic comorbidities. Subgroup analyses suggest that the strength of this association varies according to country, study design, and sepsis diagnostic criteria, underscoring the heterogeneity across existing studies.

**Systematic review registration:**

Registered with Prospero with registration number CRD420261279012.

## Background

Sepsis is a potentially life-threatening condition characterized by organ dysfunction resulting from a dysregulated immune response to infection. It ranks among the most common reasons for admission to intensive care units (ICUs) worldwide and represents a significant public health issue contributing to mortality (1, 2). According to the Global Burden of Disease (GBD) study, approximately 48.9 million cases of sepsis occurred worldwide in 2021, with related deaths reaching 11 million—accounting for nearly 20% of all global deaths that year (3). Despite significant advances in recent years in pathogen diagnosis, antimicrobial therapy, and organ function support, the mortality rate from sepsis remains high. Its complex pathogenesis, diverse clinical manifestations, and significant inter-patient variability make prognostic assessment and risk stratification ongoing focal points in clinical practice and research (4, 5).

Diabetes mellitus (DM) is a metabolic disorder characterized by chronic hyperglycemia, primarily resulting from insufficient insulin secretion or impaired insulin action (6). The global prevalence of DM has been steadily rising in recent years. According to a 2024 report by the International DM Federation (IDF), approximately 540 million adults worldwide have DM, with this number projected to exceed 700 million by 2045 (7). Patients with DM face not only risks of microvascular and macrovascular complications but also heightened susceptibility to infectious diseases (8). Research indicates that DM compromises immune defense through multiple pathways, including reduced neutrophil chemotaxis, impaired phagocytosis, abnormal cytokine release, and diminished antibody production. Furthermore, chronic hyperglycemia promotes pathogen proliferation and alters the host microbiome barrier, thereby increasing infection incidence and severity (9, 10).

Following infection, patients with DM exhibit distinct differences in inflammatory responses and immune regulation compared to non-diabetic individuals. On one hand, the hyperglycemic state associated with DM exacerbates organ damage in sepsis by enhancing the release of inflammatory mediators and oxidative stress reactions (11). On the other hand, some studies suggest that the chronic low-grade inflammation and immunosuppression prevalent in DM may partially mitigate acute inflammatory storms, thereby reducing early mortality risks in sepsis (12). This double-edged sword effect of immunity renders the relationship between DM and sepsis complex and contradictory.

In clinical practice, the coexistence of DM and sepsis is common. Studies indicate that among ICU-admitted patients with sepsis, the prevalence of DM can reach 20–30% (13). However, current clinical guidelines lack targeted recommendations for managing sepsis in patients with DM. Some studies suggest that patients with DM face higher mortality risks following infection (14), while others report opposite findings, indicating lower early mortality rates in diabetic patients with sepsis (15). Whether DM constitutes an independent risk factor for poor sepsis outcomes remains controversial. Given the limited sample sizes, significant heterogeneity among study populations, and lack of statistical significance in some previous findings, a systematic review and meta-analysis is necessary to comprehensively evaluate existing evidence. This study aims to clarify the overall direction and magnitude of the effect of DM on mortality risk in patients with sepsis.

## Methods

This systematic evaluation and meta-analysis strictly followed the PRISMA (Preferred Reporting Items for Systematic Reviews and Meta-Analyses) guidelines (16). Registered with Prospero with registration number CRD420261279012.

### Literature retrieval

A systematic search was conducted across PubMed, Embase, Web of Science, and the Cochrane Library databases from their inception to October 20, 2025, to identify all studies examining the association between DM and mortality risk in patients with sepsis. The search strategy combined Medical Subject Headings (MeSH) terms with free-text keywords, with the core query defined as: (“Sepsis” OR “Septic shock” OR “Severe sepsis” OR “Bacteremia”) AND (“Diabetes” OR “Diabetes mellitus” OR “Type 2 diabetes” OR “Type 1 diabetes” OR “Hyperglycemia”) AND (“Mortality” OR “Death” OR ‘Survival’ OR “Prognostic”). Additionally, relevant reviews, references, and conference abstracts were manually searched to supplement any omitted literature. All searches were restricted to English-language publications without limitations on region, study type, or publication date. The specific search strategy is detailed in Supplementary Table S1.

### Inclusion and exclusion criteria

Inclusion Criteria:

1. Study subjects were adults (≥18 years) with confirmed sepsis.
2. Exposure factors were DM or documented glucose metabolism abnormalities.
3. The control group comprised sepsis patients without DM.
4. Studies reporting quantitative associations between DM and mortality due to sepsis.
5. Study designs including cohort studies, case–control studies.

Exclusion Criteria:

1. Non-human studies, case reports, reviews, commentaries, or conference abstracts.
2. Outcome data failing to clearly distinguish between diabetic and non-patients with DM.
3. Studies with small sample sizes (n < 20) or incomplete data.
4. Duplicate publications; only the study of higher quality or with more complete data was retained.
5. Studies with outcomes other than mortality (incidence rates, length of hospital stays).

### Data extractions

Two authors independently screened the literature for inclusion by importing the literature into endnote according to the literature inclusion and exclusion criteria, the final included studies were used for data extraction using excel software and if there was a dispute about the literature screening then it would be discussed, or a third person would be sought to adjudicate. The extracted data contained basic characteristics of the study (first author, year of publication, country, study design), basic characteristics of the population (sample size, gender, mean age) and types of DM, mortality, diagnosis of sepsis.

### Risk of bias

The Newcastle-Ottawa Scale (NOS) (17) was used in this study to evaluate the quality of the included observational studies. The scale is categorized into three main dimensions based on the type of study (cohort study or case–control study): selection of study subjects (0–4 points), comparability of comparison groups (0–2 points) and outcome measures (0–3 points) out of a possible 9 points. A score of ≥7 is considered high quality, 5–6 is considered moderate quality and ≤4 is considered low quality. The two evaluators scored independently, and any disagreements in scoring were resolved through negotiation or referred to a third party for validation.

### Statistical analysis

All statistical analyses were performed using Stata 15.0 software. This study employed a binary data analysis approach, with patient mortality as the dichotomous outcome. For each included study, the number of deaths and total sample size in both the diabetic and non-diabetic groups were extracted. The relative risk (RR) and its 95% confidence interval (95% CI) were calculated to assess the association between DM and the risk of death from sepsis. Heterogeneity was assessed via Cochran’s Q test and the I^2^ statistic. A fixed-effects model was employed when I^2^ < 50%, indicating low heterogeneity; a random-effects model was used when I^2^ ≥ 50%, signaling substantial heterogeneity warranting further investigation into its sources. Subgroup analyses were conducted by country, study design, diagnosis of sepsis, mortality to explore potential differences in effects across groups. Sensitivity analyses were performed to assess result robustness by sequentially excluding individual studies to observe outcome stability, thereby evaluating each study’s impact on overall results. Finally, funnel plots and Egger’s test were used to assess publication bias, detecting any asymmetry that might indicate bias in the included studies, if the funnel plot is asymmetric, further assessment of the stability of the results will be conducted using trim and fill.

## Results

### Literature search results

**Figure 1:**
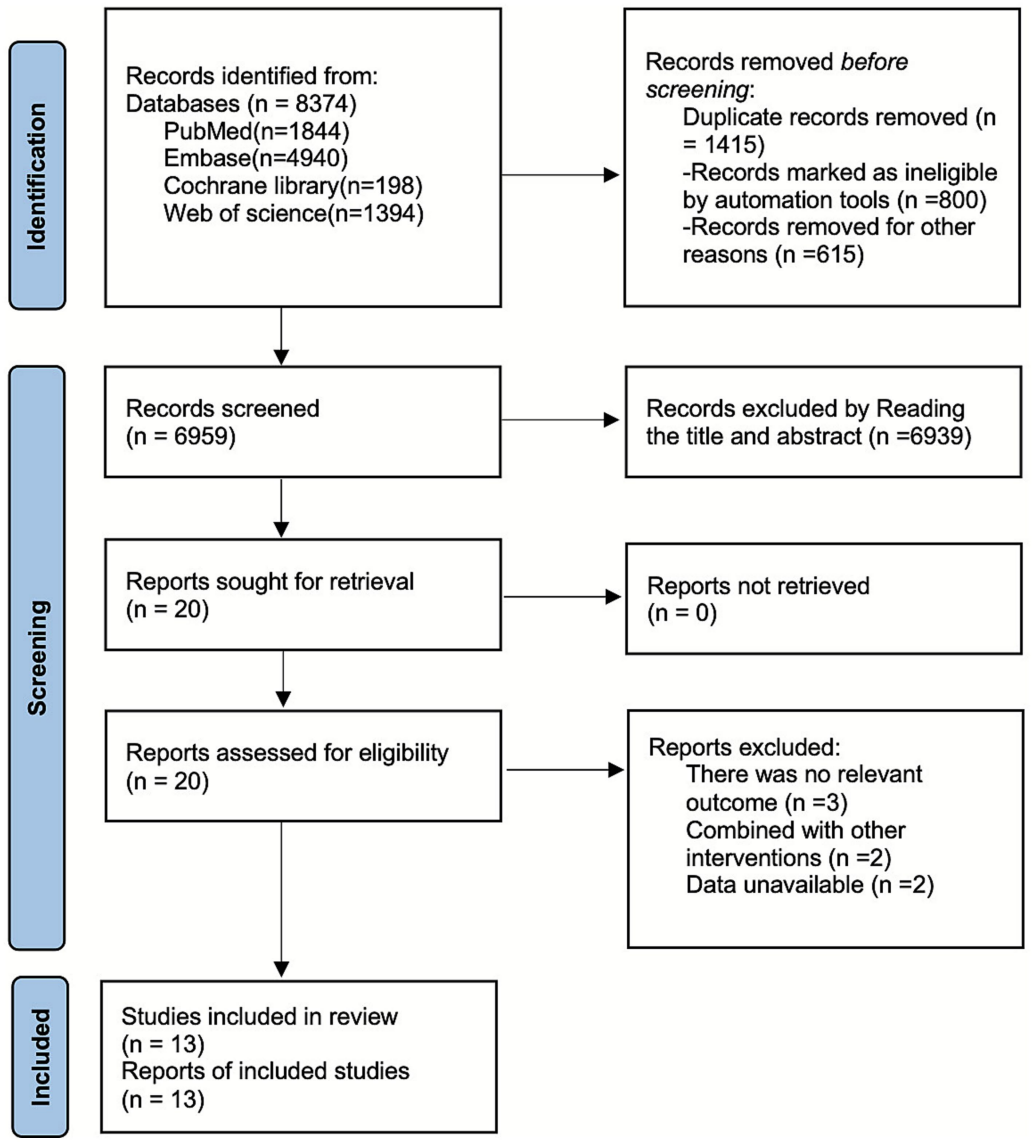
Literature search flow chart.

As shown in Figures 1, a total of 8,374 articles were retrieved from PubMed (*n =* 1,844), Embase (*n =* 4,940), the Cochrane Library (*n =* 198), and Web of Science (*n =* 1,394). After excluding 1,415 studies, 6,939 were discarded based on title and abstract review, and 7 were excluded after full-text assessment. Ultimately, 13 articles (18–30) were included.

### Basic characteristics of the included literature

A total of 13 articles were included in the analysis, involving 1,209,263 patients with sepsis across 12 cohort studies and 1 case–control study. Three studies primarily originated from China, two from Greece, two from the United States, and two from the Netherlands. The age range of participants was approximately 59–75 years. Sepsis diagnosis adhered to either the Sepsis-3 criteria or Sepsis-2 criteria. Key baseline characteristics are summarized in Table 1.

**Table 1:** Table of basic characteristics.

study	year	country	study design	sample szie	gender(M/F)	mean age(years)	Types of diabetes	SOFA scores	Diagnosis of sepsis	mortality
Abe	2020	Japan	cohort study	619	371/248	75	Unclassified	8.5	Sepsis-2 criteria	In-hospital
Akinosoglou	2021	Greece	cohort study	812	370/442	76.2	type 2 diabetes	NR	Sepsis-3 criteria	28 day
Angriman	2024	Canada	cohort study	503,455	230,079/273376	73.3	type 1 and type 2 diabetes mellitus	NR	Sepsis-2 criteria	28dya/90 day
Chang	2012	China	cohort study	16,497	10,133/6364	67.8	type 2 diabetes	NR	Sepsis-3 criteria	90 day
Chao	2017	China	cohort study	6,165	3337/2828	66	Unclassified	NR	Sepsis-3 criteria	In-Hospital Mortality
Lin	2021	China	cohort study	12,321	6493/5828	67.1	Unclassified	5	Sepsis-3 criteria	28 day/In-hospital mortality/ICU mortality
Moutzouri	2008	Greece	cohort study	64	34/30	63	Unclassified	NR	Sepsis-3 criteria	Mortality
Sathananthan	2020	USA	cohort study	1,698	988/710	63.5	type 2 diabetes	NR	Sepsis-3 criteria	Mortality
Schuetz	2011	USA	cohort study	7,754	3872/3882	59	Unclassified	NR	NR	Mortality
Stegenga	2010	Netherlands	cohort study	830	481/349	60.6	Unclassified	2.68	NR	28dya/90 day
van	2017	Netherlands	cohort study	41,492	23,651/17841	69.5	Unclassified	NR	Sepsis-2 criteria	In-hospital/ICU/90 day
Venot	2015	France	case–control	1,064	687/377	67.2	Unclassified	NR	NR	ICU
Zohar	2010	Israel	cohort study	1,527	667/860	67	Unclassified	NR	Sepsis-3 criteria	28dya/90 day/In-hospital

### Quality evaluation

This study employed the NOS scoring system for evaluation, with 8 studies scoring 8 points and 5 studies scoring 9 points. The overall article quality was rated as high. Detailed scores are presented in Table 2.

**Table 2:** NOS score results table.

Case control									
Study	Is the case definition adequate?	Representativeness of the cases	Determination of control group	Definition of Controls	Comparability of cases and controls based on the design or analysis	Ascertainment of exposure	Same method of ascertainment for cases and controls	Non response	Total scores
Venot2015	*	*	*	*	**	*	*	*	9

Cohort study									
Study	Representativeness of the exposed group	Selection of non-exposed groups	Determination of exposure factors	Identification of outcome indicators not yet to be observed at study entry	Comparability of exposed and unexposed groups considered in design and statistical analysis	design and statistical analysis	Adequacy of the study’s evaluation of the outcome	Adequacy of follow-up in exposed and unexposed groups	Total scores
Abe 2020	*	*	*	*	**	*	*	*	9
Akinosoglou 2021	*	*	*	*	*	*	*	*	8
Angriman 2024	*	*	*	*	*	*	*	*	8
Chang 2012	*	*	*	*	**	*	*	*	9
Chao 2017	*	*	*	*	*	*	*	*	8
Lin 2021	*	*	*	*	*	*	*	*	8
Moutzouri 2008	*	*	*	*	**	*	*	*	9
Sathananthan 2020	*	*	*	*	*	*	*	*	8
Schuetz2011	*	*	*	*	*	*	*	*	8
Stegenga 2010	*	*	*	*	**	*	*	*	9
van 2017	*	*	*	*	*	*	*	*	8
Zohar 2010	*	*	*	*	*	*	*	*	8

## Results of meta-analysis

### Association between DM and mortality due to sepsis

**Figure 2:**
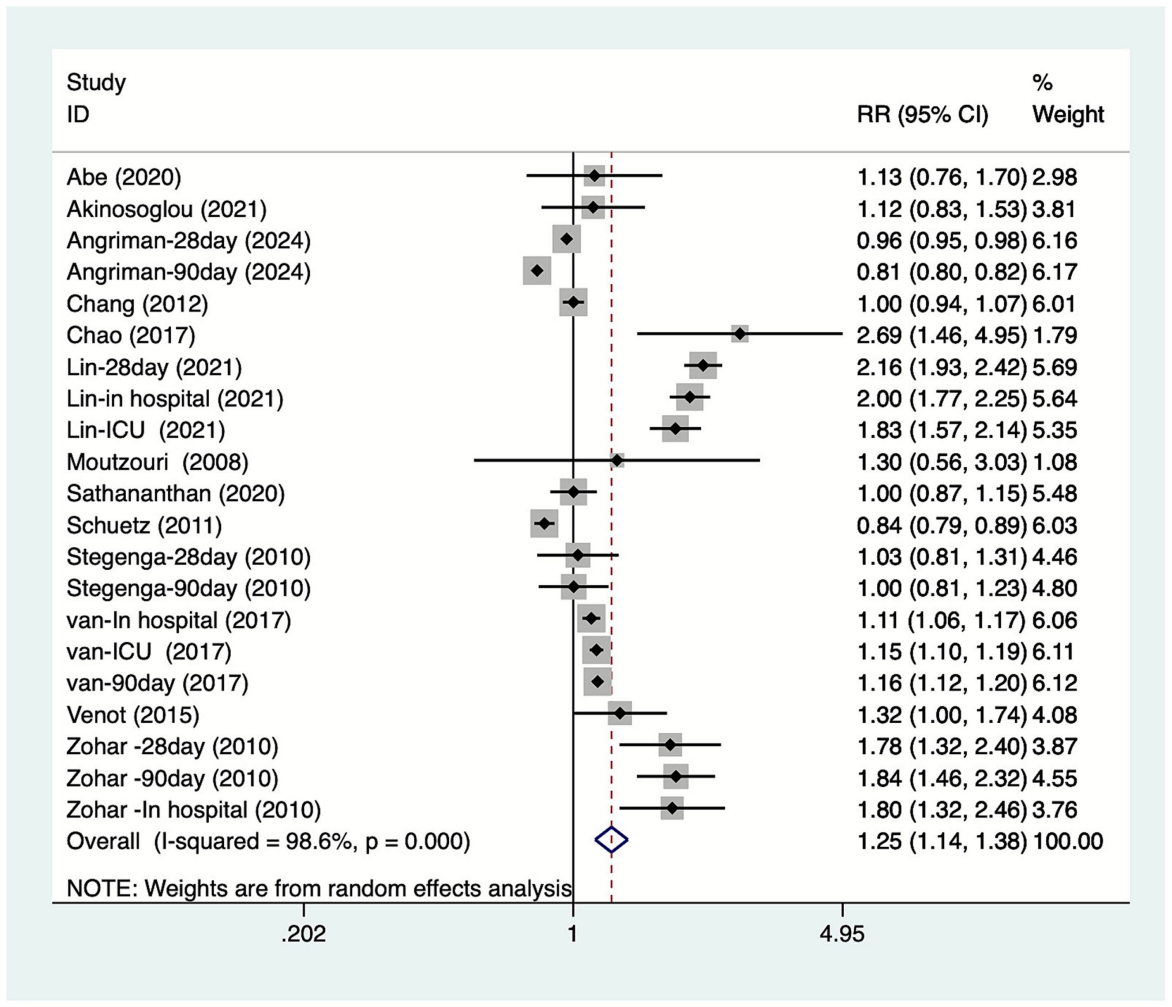
Forest plot of meta-analysis of association between diabetes and mortality due to sepsis.

Thirteen articles discussed the association between DM and mortality due to sepsis. Among these, Angriman et al. (20) reported 28-day and 90-day mortality rates; Lin et al. (23) reported 28-day, ICU, and in-hospital mortality rates; Stegenga et al. (27) reported 28-day and 90-day mortality rates; van Vught et al. (28) reported ICU, 90-day, and in-hospital mortality rates, while Zohar et al. (30) reported 28-day and 90-day in-hospital mortality rates. Heterogeneity testing (I^2^ = 98.6%, *p* = 0.001) was performed using a random-effects model. Results (Figure 2) suggest DM increase mortality in patients with sepsis [RR = 1.25, 95% CI (1.14, 1.38)]. Due to substantial heterogeneity, sensitivity analysis was conducted by sequentially excluding studies. Results (Supplementary Figure S1) indicate stable findings unaffected by individual studies.

### Subgroup analysis results

**Figure 3:**
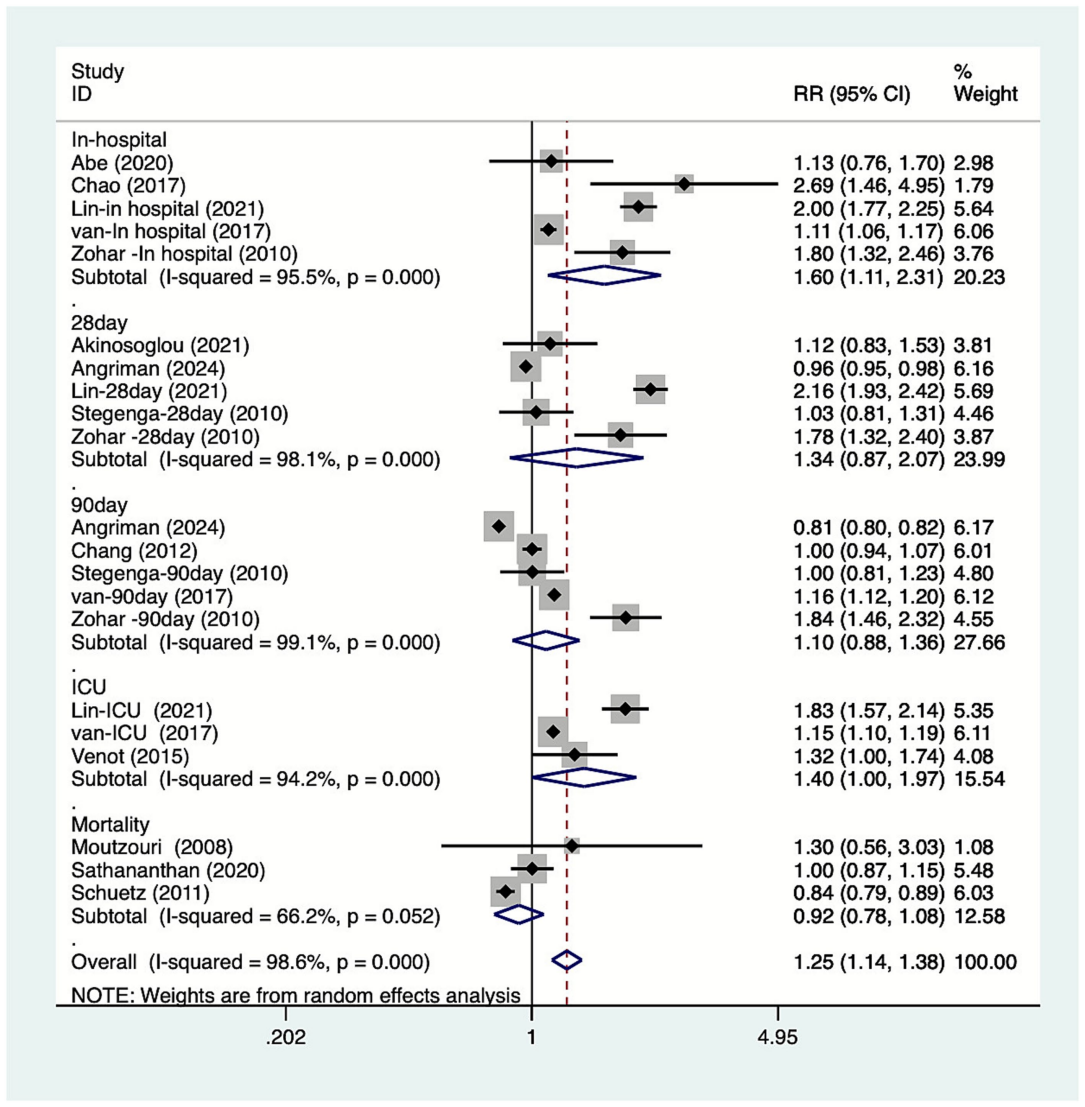
Meta-analysis of the association between diabetes and mortality due to sepsis subgroup forest plots of mortality types.

**Figure 4:**
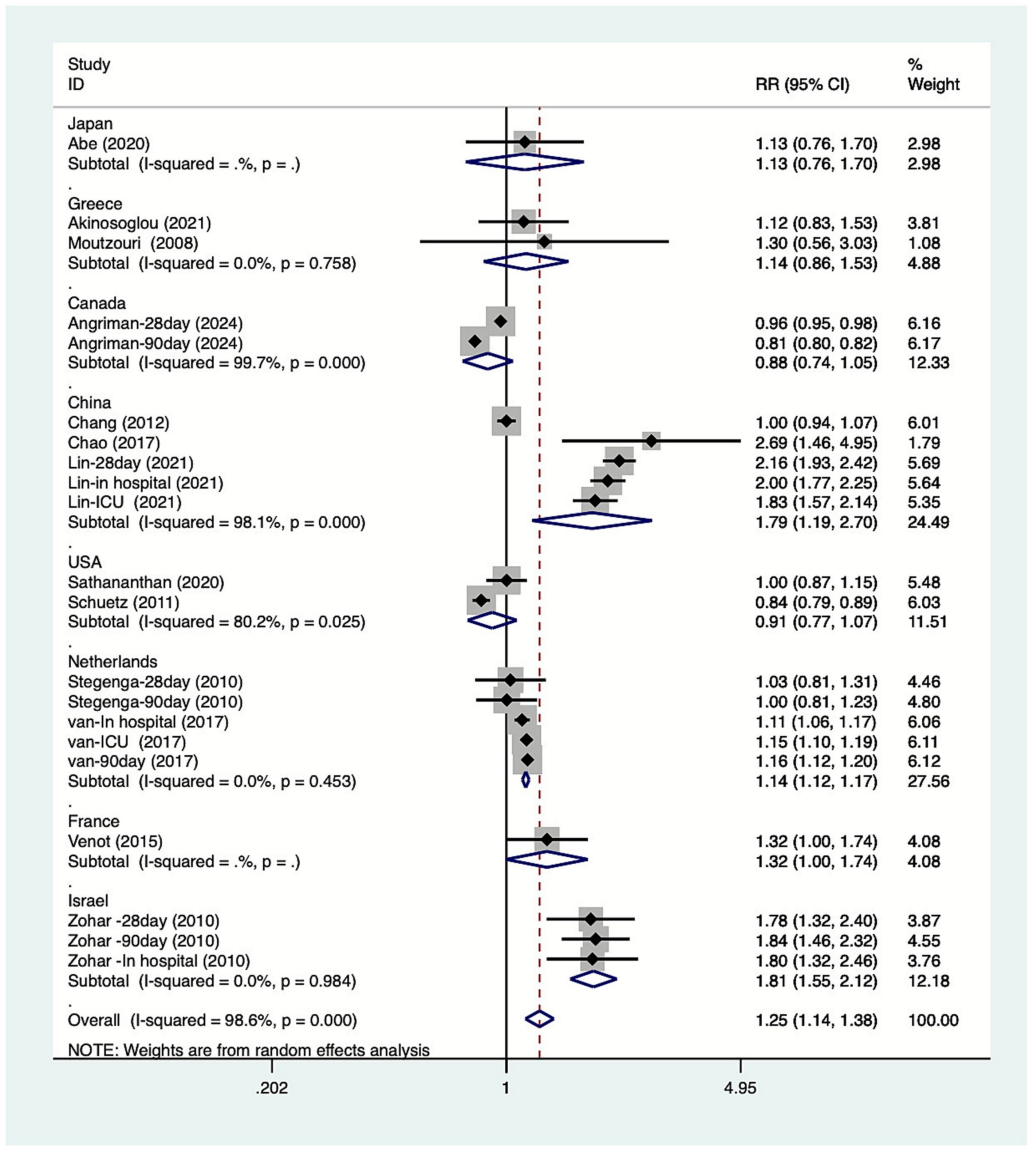
Meta-analysis of the association between diabetes and mortality due to sepsis subgroup forest plots of country.

**Figure 5:**
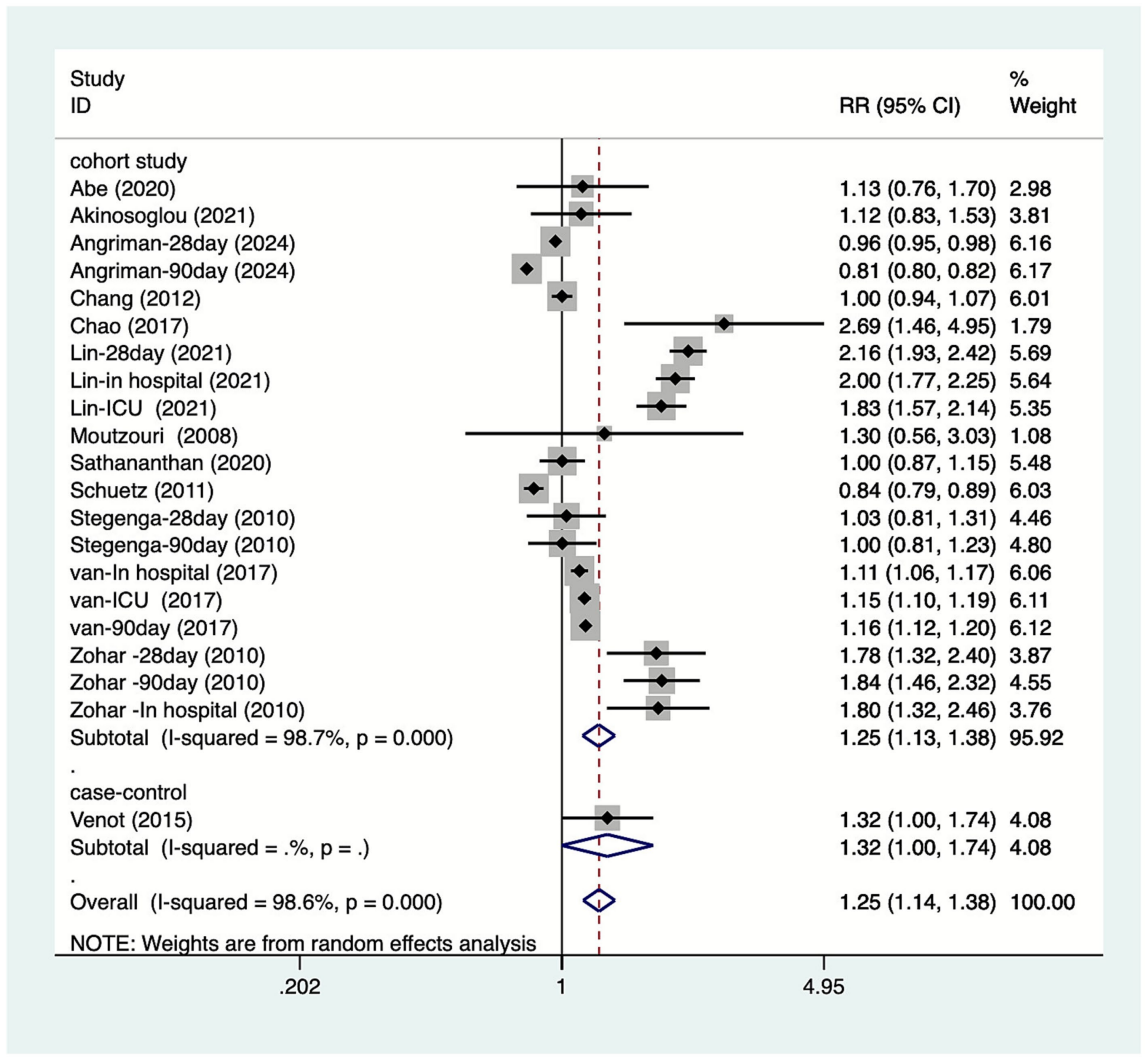
Meta-analysis of the association between diabetes and mortality due to sepsis subgroup forest plots of study design.

**Figure 6:**
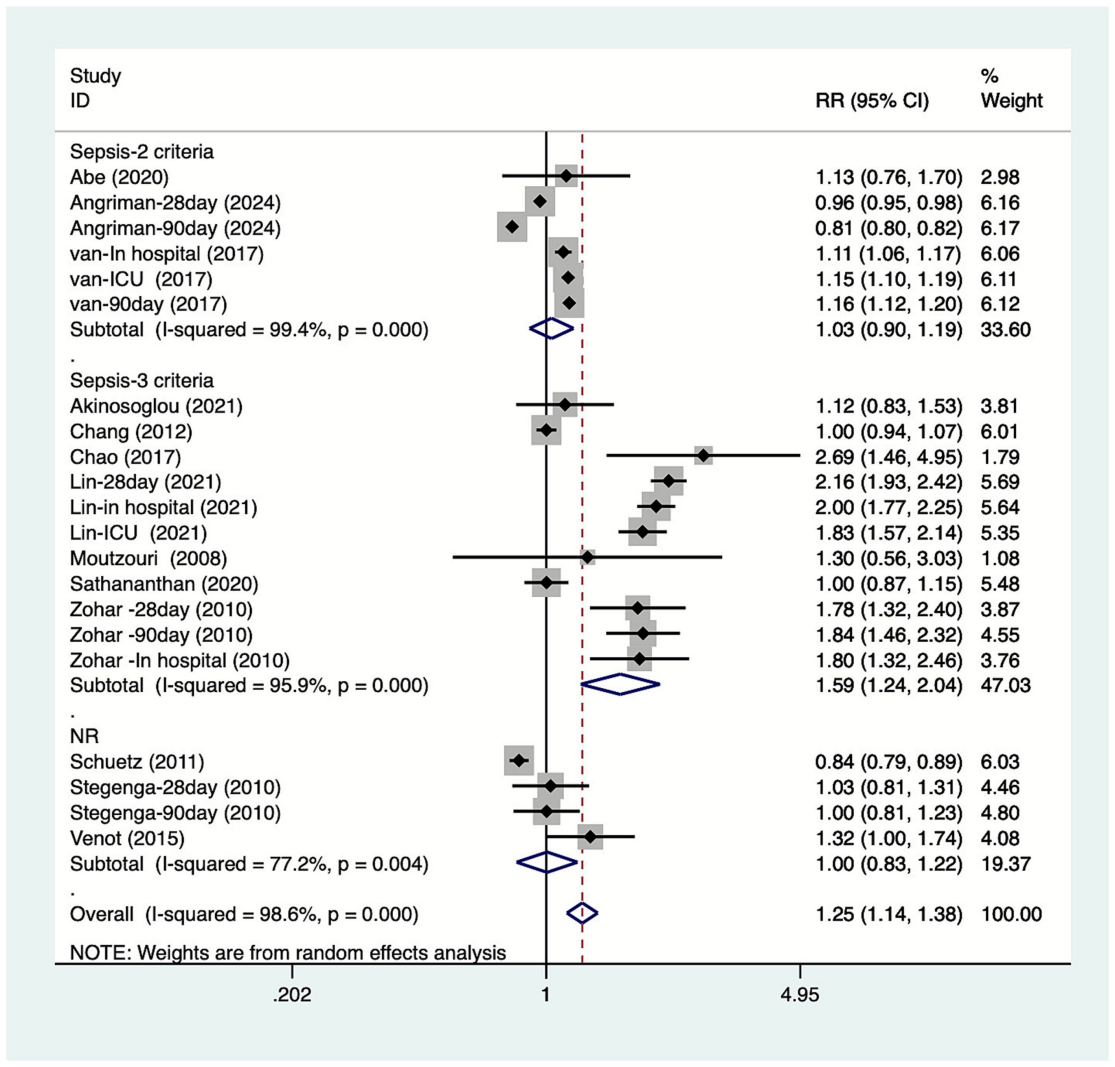
Meta-analysis of the association between diabetes and mortality due to sepsis subgroup forest plots of diagnosis of sepsis.

The study conducted subgroup analyses based on the type of mortality. The results (Figure 3) indicated that DM increase in-hospital mortality among patients with sepsis [RR = 1.60, 95% CI (1.11, 2.31), *n =* 5], while it had no significant effect on 28-day mortality [RR = 1.34, 95% CI (0.87, 2.07), *n =* 5], 90-day mortality [RR = 1.10, 95% CI (0.88, 1.36), *n =* 5], or ICU mortality [RR = 1.40, 95% CI (1.00, 1.97), *n =* 3]. The study conducted subgroup analyses based on the country. The results (Figure 4) indicated that DM increase the mortality of patients with sepsis in China [RR = 1.79, 95% CI (1.19, 2.70), *n =* 5], the Netherlands [RR = 1.14, 95% CI (1.12, 1.17), *n =* 5], and Israel [RR = 1.81, 95% CI (1.55, 2.12), *n =* 3]. The study conducted subgroup analyses based on the study design. The results (Figure 5) indicated that DM increase cohort study [RR = 1.25, 95%CI (1.13, 1.38)] mortality among patients with sepsis. The study conducted subgroup analyses based on the diagnosis of sepsis. The results (Figure 6) indicated that DM increase Sepsis-3 criteria [RR = 1.59, 95%CI (1.24, 2.04), *n =* 12] mortality among patients with sepsis.

### Publication bias

This study employed funnel plots and Egger’s test to assess publication bias. Results (Supplementary Figure S2) indicate asymmetry in the funnel plot for “Association Between DM and Mortality due to sepsis,” with an Egger’s *p*-value of 0.003, suggesting a high likelihood of publication bias. Consequently, trim-and-fill methods were applied to evaluate result stability. Results (Supplementary Figure S3) demonstrate that despite the presence of publication bias, the findings remain robust.

## Discussion

This study is a meta-analysis examining the relationship between DM and mortality risk in patients with sepsis. Based on the combined results of 13 studies, we found that DM is significantly associated with increased mortality risk in patients with sepsis, but this association is not uniform across all individuals. The risk may vary depending on factors such as glycemic control, the presence of comorbidities, and the severity of sepsis, underscoring the importance of individualized risk assessments in patients with DM with sepsis. These findings provide further evidence of DM on the prognostic of patients with sepsis and underscore that individuals with DM may face a higher risk of mortality during sepsis. Our findings align with previous research (31), further confirming DM adverse prognostic role in sepsis. Patients with DM typically experience chronic hyperglycemia, a state that compromises the immune system—particularly by suppressing neutrophil function (32). This reduces neutrophil chemotaxis, phagocytic capacity, and bactericidal activity, making patients with DM more susceptible to severe complications during bacterial infections (33). DM also causes endothelial dysfunction and promotes the release of inflammatory mediators, further exacerbating the systemic inflammatory response syndrome triggered by sepsis. The combination of these factors may represent a potential mechanism linking DM to increased mortality risk in sepsis (34).

The primary finding of this study is that patients with DM exhibit a significantly increased risk of mortality during sepsis compared to patients without DM. Through subgroup analyses, we further explored the impact of DM on different types of mortality, including in-hospital mortality, 28-day mortality, 90-day mortality, and ICU mortality. Results indicate that DM significantly increases in-hospital mortality among patients with sepsis, reflecting high mortality during the hospital admission period, but does not significantly affect ICU mortality. This finding suggests that DM exerts a greater influence on mortality risk during the acute phase of sepsis within the hospital, particularly during the early stages of hospitalization.

The differential impact of DM on mortality risk in patients with sepsis may be closely associated with poor glycemic control, complications, and treatment approaches during hospitalization (33). Patients with DM with sepsis may face more complex clinical scenarios. For instance, hyperglycemia may impair immune function, while comorbidities such as advanced age, cardiovascular disease, and renal insufficiency may further exacerbate the patient’s condition (35). Additionally, infection control in patients with DM may be affected by DM management, particularly during hospitalization when clinical status can change rapidly. Consequently, patients with DM experience a significantly increased risk of in-hospital mortality (36).

In addition to mortality types, we conducted subgroup analyses to assess the impact of different countries, study designs, and sepsis diagnostic criteria on the association between DM and mortality risk. Subgroup analysis results indicated that the effect of DM on mortality due to sepsis varied significantly across countries, particularly in studies from China, the Netherlands, and Israel, where patients with DM exhibited higher mortality risk. The results from China showed an RR of 1.79, the Netherlands 1.14, and Israel 1.81, suggesting that regional differences may be related to variations in healthcare standards, DM management approaches, and sepsis treatment protocols. Differences in DM management, healthcare resources, and clinical diagnosis and treatment across countries may lead to varying degrees of impact of DM on mortality due to sepsis (37). Regarding study design, our subgroup analysis confirmed that cohort studies consistently demonstrated elevated mortality risk in patients with DM, further validating the significant association between DM and mortality due to sepsis. This aligns with most observational studies, underscoring DM potential role in poor prognostic among patients with sepsis. Additionally, subgroup analyses revealed that the impact of DM on mortality due to sepsis was more pronounced in studies using Sepsis-3 criteria for diagnosis. This may stem from Sepsis-3’s more precise definition of sepsis, which better captures DM influence on mortality risk. The Sepsis-3 criteria provide a clearer definition of sepsis and offer a more accurate pathophysiological framework, potentially revealing the true impact of DM on mortality in patients with sepsis (38). Heterogeneity was observed across subgroups, particularly in studies from different countries and using different diagnostic criteria. The clinical meaning of this heterogeneity may reflect variations in healthcare quality, treatment regimens, and diagnostic precision. For example, the broader confidence intervals observed in some subgroups might indicate that differences in local practices, patient populations, and study methodologies contribute to the variability in the association between DM and mortality due to sepsis.

### Strengths and limitations

The strengths of this study lie in its systematic and comprehensive approach. First, it includes a broad patient population and diverse study designs, ensuring high representativeness and broad external validity of the findings. By employing random-effects models to synthesize results across studies, we account for heterogeneity more effectively, yielding robust conclusions. Second, we conducted detailed subgroup analyses to explore the impact of various factors—including different types of mortality, geographic regions, study designs, and sepsis diagnostic criteria—on the association between DM and mortality risk. This approach provided a deeper understanding of DM’s role in sepsis pathogenesis and offered more targeted guidance for clinical practice. Finally, sensitivity analyses were performed to assess the influence of individual studies on the overall results, further enhancing the reliability and robustness of our findings.

Although this study provides valuable conclusions, several limitations remain. First, despite using a random-effects model to pool results, the inclusion of observational studies precludes complete elimination of potential confounding factors. For instance, factors such as glycemic control in patients with DM, underlying comorbidities (cardiovascular disease, kidney disease), infection type, and sepsis severity may influence mortality rates. However, most studies did not perform detailed adjustments for these variables. The lack of sufficient multivariable analysis prevented us from further controlling these potential confounders, potentially affecting the accuracy of the findings and the ability to draw causal inferences. Second, substantial heterogeneity existed between studies (I^2^ = 98.6%), likely attributable to differences in patient populations, study designs, diagnostic criteria, and other variables. Although sensitivity analyses and subgroup analyses adjusted for some factors, heterogeneity remains a limitation of this review. Third, most included studies did not perform detailed stratified analyses based on DM type (type 1 or type 2 diabetes), disease duration, or treatment modalities. Consequently, we could not evaluate the specific impact of these factors on mortality due to sepsis risk. Finally, publication bias within the studies may also compromise the accuracy of conclusions, as studies reporting non-significant associations might have been excluded from the analysis, potentially leading to an overestimation of the results.

### Clinical significance and future directions

This study demonstrates an association between DM and increased mortality in patients with sepsis, with a particularly evident relationship for in-hospital mortality. However, the findings should not be interpreted as suggesting that all patients with DM face uniformly high mortality risk during sepsis. Rather, we emphasize the importance of individualized risk assessment in patients with DM with sepsis. The impact of DM on mortality risk may vary significantly depending on factors such as the type of DM (type 1 vs. type 2), the degree of glycemic control, the presence of comorbidities, and the management strategies employed during hospitalization. Thus, it is crucial to consider these factors when evaluating the prognostic of patients with DM in sepsis, rather than assuming a higher mortality risk universally.

These findings contribute to the existing evidence base by highlighting DM as an important comorbidity that should be considered as part of a personalized risk stratification approach in septic patients. However, due to the observational nature of the included studies, definitive conclusions regarding optimal clinical management strategies for patients with DM with sepsis cannot be drawn. Future research should focus on elucidating how DM-related characteristics, including glycemic control and comorbidities, influence sepsis outcomes, and how these factors can be incorporated into individualized management strategies.

## Conclusion

This meta-analysis indicates that DM is significantly associated with an increased risk of mortality in patients with sepsis, particularly with respect to in-hospital mortality. However, the study did not investigate whether deaths were due to failure of monitoring, and therefore, the need for enhanced monitoring cannot be conclusively recommended based solely on the results. The observed association may be partially explained by DM-related pathophysiological mechanisms, such as immune dysfunction, dysregulated inflammatory responses, and the presence of chronic comorbidities. Subgroup analyses suggest that the strength of this association varies according to country, study design, and sepsis diagnostic criteria, highlighting the heterogeneity across existing studies. Overall, these findings emphasize the importance of considering DM as a relevant prognostic factor in sepsis research. Further high-quality prospective studies are needed to clarify causal relationships and to determine whether DM-related factors represent modifiable targets for improving outcomes in patients with sepsis.

## Data Availability

The original contributions presented in the study are included in the article/Supplementary material, further inquiries can be directed to the corresponding author.
