# Supplementary material for: Diabetes mellitus and mortality in patients admitted to ICU with sepsis: a meta-analysis
Source: Front Med (Lausanne). 2026 Jan 21;13:1743706. doi: 10.3389/fmed.2026.1743706 (PMC12867908; doi:10.3389/fmed.2026.1743706)
Supplement: Supplementary file 1 [file Data_Sheet_1.DOCX]

Table S1 specific search strategy

((("Diabetes Mellitus"[Mesh]) OR (Diabetes Mellitus[Title/Abstract])) AND (("Sepsis"[Mesh]) OR ((((((((((((((((((Sepsis[Title/Abstract]) OR (Bloodstream Infection[Title/Abstract])) OR (Bloodstream Infections[Title/Abstract])) OR (Infection, Bloodstream[Title/Abstract])) OR (Septicemia[Title/Abstract])) OR (Septicemias[Title/Abstract])) OR (Blood Poisoning[Title/Abstract])) OR (Blood Poisonings[Title/Abstract])) OR (Poisonings, Blood[Title/Abstract])) OR (Poisoning, Blood[Title/Abstract])) OR (Severe Sepsis[Title/Abstract])) OR (Sepsis, Severe[Title/Abstract])) OR (Pyemia[Title/Abstract])) OR (Pyemias[Title/Abstract])) OR (Pyaemia[Title/Abstract])) OR (Pyaemias[Title/Abstract])) OR (Pyohemia[Title/Abstract])) OR (Pyohemias[Title/Abstract])))) AND (("Death"[Mesh]) OR ((((((((Death[Title/Abstract]) OR (End Of Life[Title/Abstract])) OR (End-Of-Life[Title/Abstract])) OR (Determination of Death[Title/Abstract])) OR (Near-Death Experience[Title/Abstract])) OR (Cardiac Death[Title/Abstract])) OR (Death, Cardiac[Title/Abstract])) OR (mortality[Title/Abstract])))


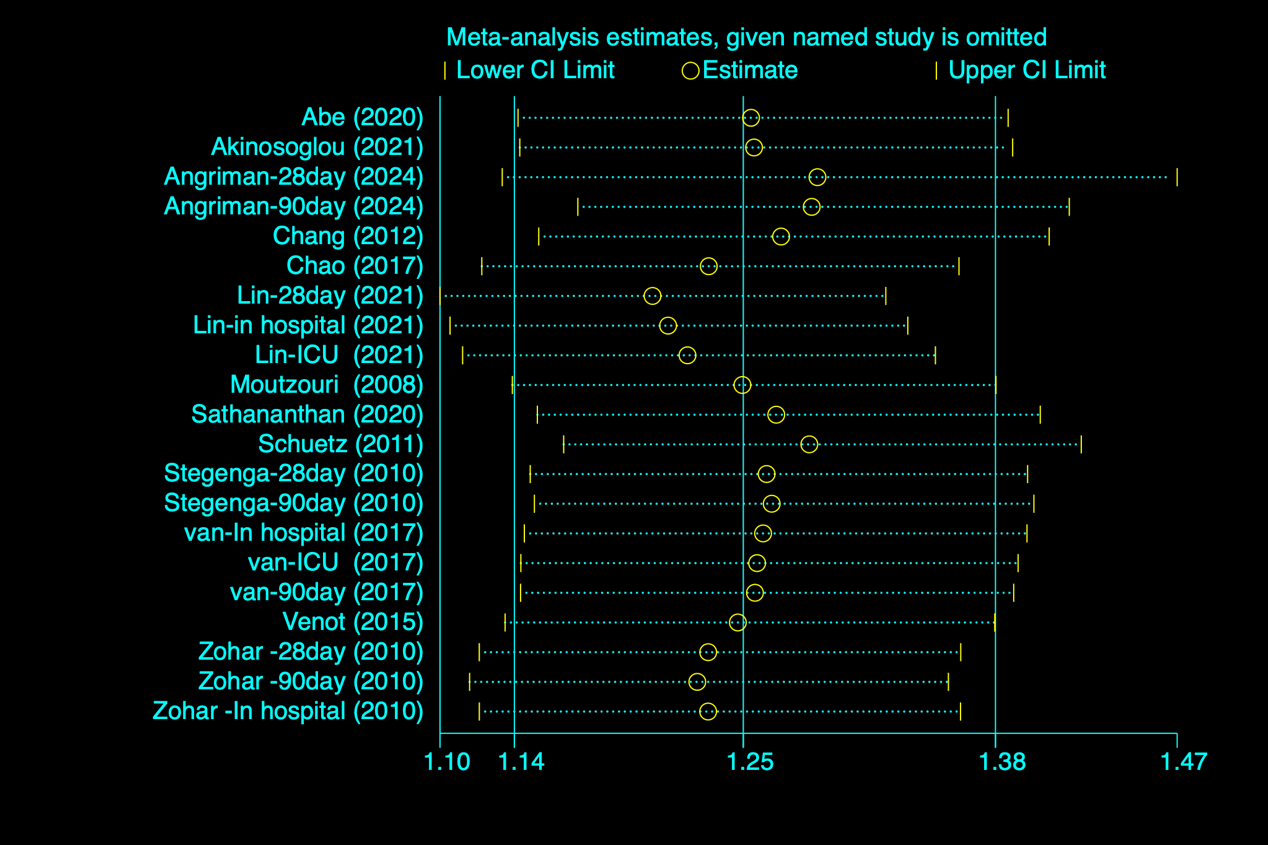


Figure S1 Results of sensitivity analysis of the Association Between Diabetes and Sepsis Mortality


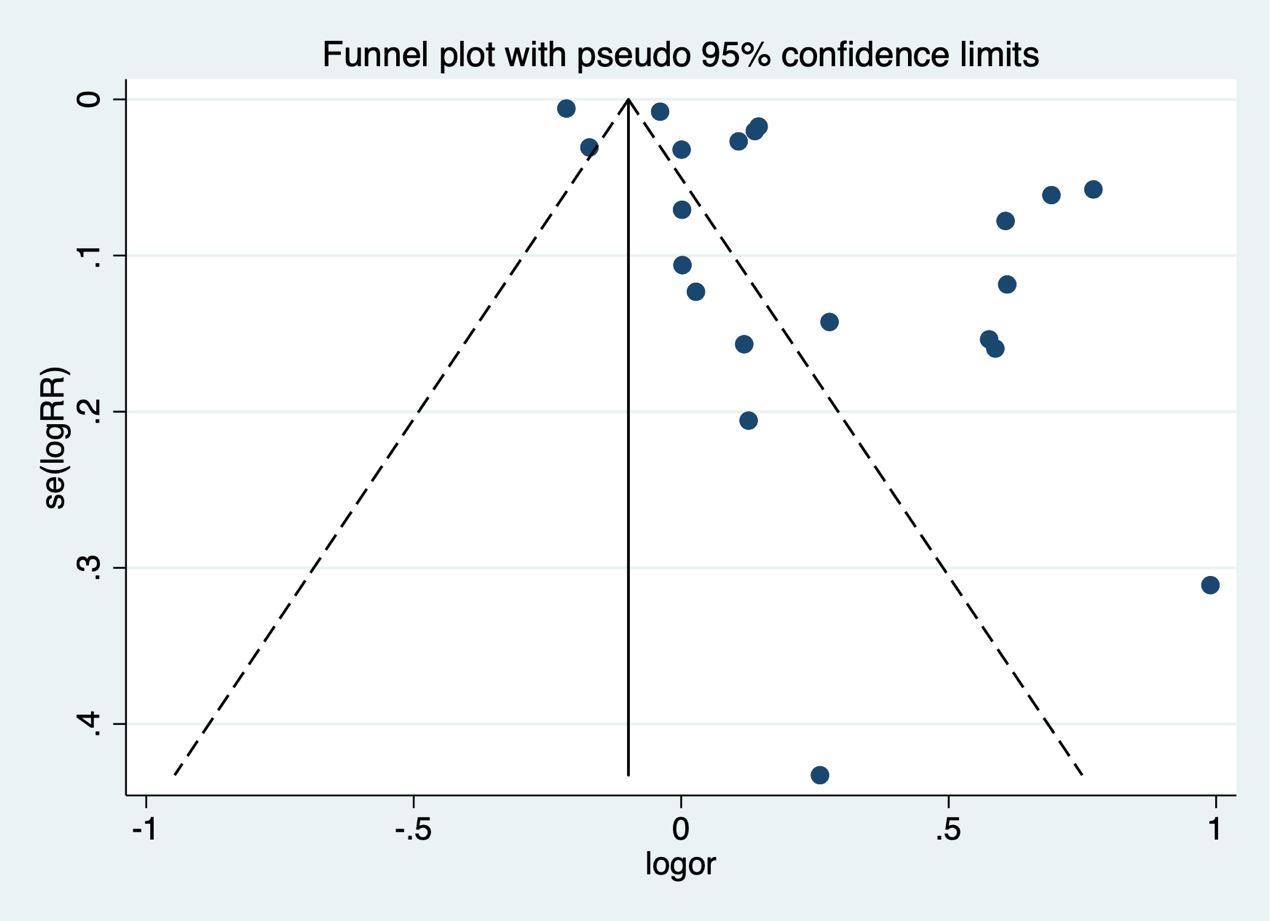


Figure S2 Funnel plot of meta-analysis of Association Between Diabetes and Sepsis Mortality


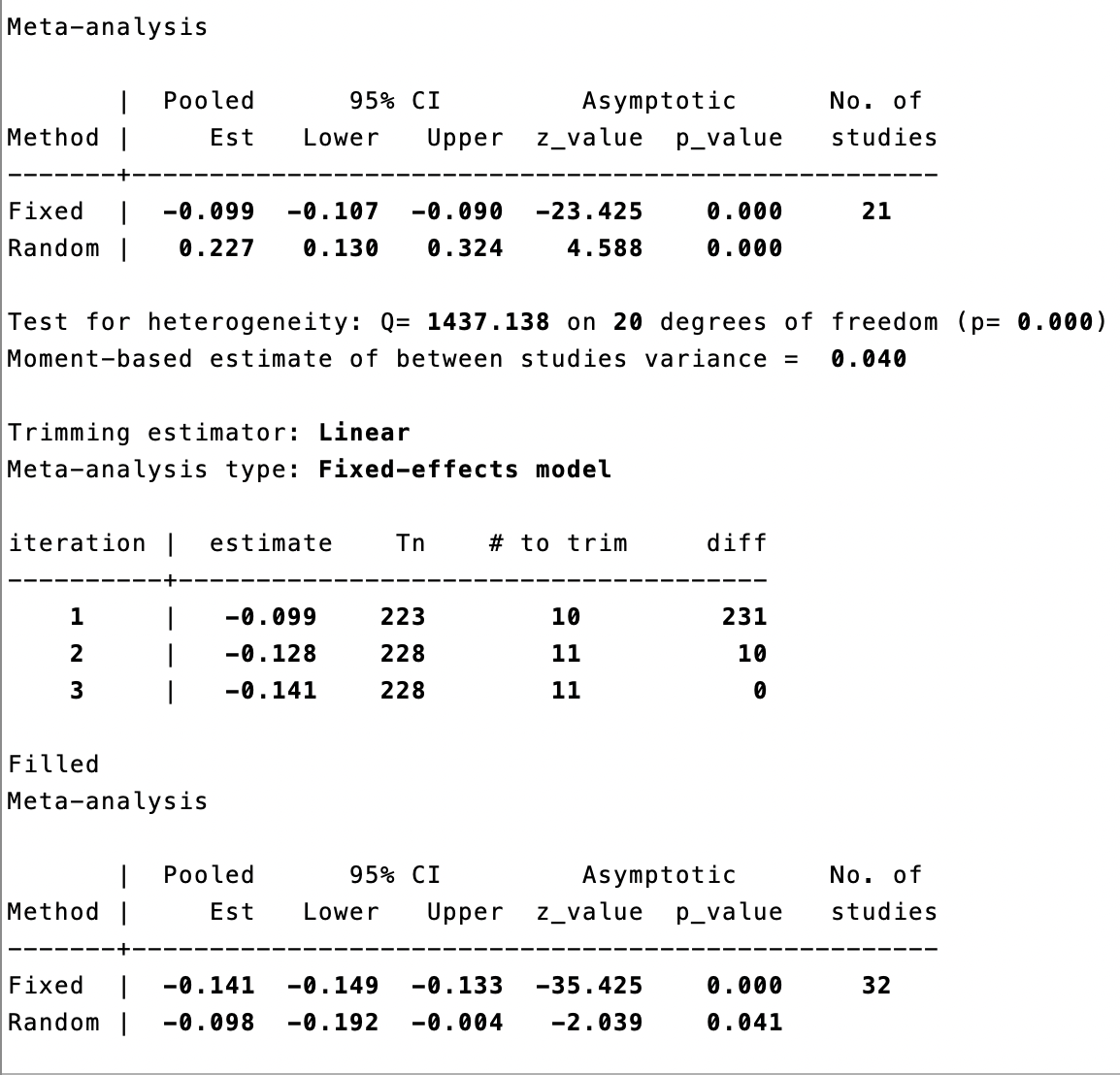


Figure S3 trim-and-fill results on the Association Between Diabetes and Sepsis Mortality
